# The Naval and Military Services as a Career

**Published:** 1908-09-05

**Authors:** 


### The Public Services.

### THE NAVAL AND MILITARY SERVICES AS A CAREER.

Members of the medical profession are fortunate in that their choice of surroundings in which to work is extremely varied. Wherever civilised man goes, and in however few numbers, he needs a doctor before long; and if he goes to fight he needs one more than ever. To a considerable proportion of each year's output of qualified medical men careers have for long been afforded by the medical departments of our national forces. Hitherto the most popular has been the Indian Medical Service, although the Royal Army Medical Corps is beginning to catch up on it fast in this respect. It is to be hoped that before long the measures which have secured this desirable result will be found operative in the case of the Navy. The first named service offers, of course, a strong monetary attraction, particularly to those electing for civil employ. The nature of the Indian climate makes this imperative, and probably a certain number of the pick of the profession will always continue to be attracted by the high pay, by the general standard of work—which must needs reach the consultant grade, and is further favourably influenced through contact with the traditions of an efficient civil service—and by the chance of influential position which an elastic bureaucratic system affords. Tropical service in the Navy, or in the British Army, while not allowed for at the same liberal rate, compares well pecuniarily, as indeed it should, with ordinary medical work at home. The average total income of the English medical man has been reckoned at £200 per annum. Now, after sixteen years, a surgeon in the Navy is earning £500 a year, is getting quarters free, and food at a very cheap rate, and can, if he likes, retire in four years' time on a pension of £1 a day. The cost of military or naval uniform, too (eighty or a hundred odd pounds) would not go far towards buying a practice.

The routine duties of a civil practitioner differ much from those of a military medical officer—not only because there is naturally much more of purely professional work involved in attending to the sick of a community, however small, than in the medical care of a physically selected body of men who are most of them single. It must be remembered that the Army Medical Department undertakes almost entire administrative control of its hospitals, this course having been found advisable from every point of view. Lack of clinical opportunity and prepossession with administrative work—both of them inevitable and the latter also highly necessary—have in the past been productive in some instances of a certain slackness over professional matters, with a consequent loss of prestige; although it is rather overlooked that in this country hygiene and preventive medicine may almost be said to have taken their rise from Netley. It is only necessary to mention in this connection the names of de Chaumont and Parkes, and latterly those of Wright, Bruce, and Leishman. Nowadays, thanks partly to a wise improvement in the conditions of medical service, partly to the advance of tropical medicine, a healthier spirit is being manifested. The co-operation of naval and military medical officers has recently had a triumph at Malta in the control of Mediterranean fever; the system of specialist study, inaugurated by medical authorities of the home Army, is being taken up also by the Indian Medical Service, which may furnish others to follow in Professor Ross's footsteps; if any entrants to the Royal Army Medical Corps expect an easy time they find themselves quickly undeceived at the College at Millbank. In all branches the number is growing of those who aim at reaching a high professional standard, instead of merely the position of what may be called, in Meredith's words, "a gentlemanly official."

This point leads on to the consideration of a more practical and always important one, namely, the relations between medical men and the laity—in this particular instance between the medical and the combatant or executive officers. It would be glossing things over to say that in the past these have always been ideal, and that the causes of occasional friction were only temporary ones. Indeed, two bed-rock facts of the situation are, firstly, the national, and especially, perhaps, the aristocratic, distrust of experts as authoritative advisers; and secondly, the fact that, generally speaking, the commissioned ranks of the Navy and Army are recruited from higher social strata than those obtaining with the medical profession. In all probability faults have been more on one side than on the other, and recent events have improved matters considerably. Nevertheless, it does not need much penetration to perceive the value in this connection also of the maxim, "Be good doctors."

In the Navy, where opportunities for active service are happily comparatively rare, influence counts for much in advancement; and the same holds good to a less extent of the other two services named. But after all, this only applies to the minor prizes, and can offer no bar to outstanding ability. Other more or less obvious qualifications for ordinary success are readiness and practical ability, sociability, courtesy, a strong constitution, and fondness for travel and for sport or games. London hospitals have many alumni possessing these qualities, and probably in the future more of them will be found entering the services But anyone who thinks of doing so must be prepared for expatriation. It is true that the stated amounts of leave look very liberal compared with the three weeks or a month per year which is all that most general practitioners can treat themselves to, but to enjoy such absence from duty in a temperate climate may involve heavy expense, especially to a married man. The intending candidate should also have a not easily satisfied and abiding taste for frequent change of scene, with a tolerance for the inconveniences involved; and it is better for him not to marry until he can count four or five years' service.

